# Midwifery students better approximate their self-efficacy in clinical lactation after reflecting in and on their performance in the LactSim OSCE

**DOI:** 10.1186/s41077-020-00143-z

**Published:** 2020-10-23

**Authors:** Aria Grabowski, Olivia S. Anderson, Ruth Zielinski, Melisa Scott, Lisa Hammer, Muriel Bassil, Samantha A. Chuisano, Anna Sadovnikova

**Affiliations:** 1 Department of Nutritional Sciences, School of Public Health, University of Michigan, Ann Arbor, USA; 2 School of Nursing, University of Michigan, Ann Arbor, USA; 3 University of Michigan, Ann Arbor, USA; 4 LiquidGoldConcept, Inc., Ypsilanti, USA; 5 Physician Scientist Training Program, Graduate Group in Nutritional Biology, University of California, Davis, Davis, USA

**Keywords:** Breastfeeding education, High-fidelity simulation, Lactation simulation model, Midwifery education, Observed structured clinical examination, Self-reflection, Abbreviations, LactSim Lactation simulation, OSCE Objective structured clinical exam, LSM Lactation simulation model, IBCLC International Board-Certified Lactation Consultant

## Abstract

**Background:**

Midwives are expected to support women with lactation initiation and maintenance. Midwifery students engaged in a simulation-based exercise (LactSim OSCE) where they role-played the clinician and the breastfeeding patient by wearing a high-fidelity breast model. We provided participants opportunities for reflecting in and on practice to compare their perceived self-confidence in clinical lactation skills to actual clinical performance. We also describe feasibility of implementing the LactSim OSCE with an emphasis on preparation and time spent on tasks during the OSCE.

**Methods:**

Audio-video recordings from the LactSim OSCE were viewed and assessed using a technical skills checklist by an independent rater and by the study participants as part of the self-reflection. Mixed data on participants’ self-efficacy in clinical lactation, experience with the LactSim OSCE, and self-assessment of clinical performance were collected in survey instruments and a focus group. Time spent on each component and clinical lactation skill during the LactSim OSCE was documented.

**Results:**

Immediately following the LactSim OSCE, participants’ confidence in clinical lactation was high (5.7/7), but after a guided video reflection exercise, their self-efficacy was 4.4/7. Participants spent approximately 2 of the allotted 10 min per case scenario discussing the OSCE logistics due to inadequate preparation. Participants spent approximately 2 min of the total encounter performing hands-on clinical lactation skills by touching, looking at, or using the high-fidelity breast model worn by their peer.

**Conclusion:**

We described the development and evaluation of the first simulated experience in clinical lactation with all three components of fidelity: conceptual, psychological, and physical. Multiple opportunities for reflecting on performance allowed the nurse-midwifery students to evaluate their competence in decision-making, technical, and counseling skills which resulted in a more realistic approximation of their perceived self-confidence in breastfeeding skills. Another innovation of this pilot work is the documentation of how long a learner spends on various tasks relevant to lactation support in a simulated encounter. Our findings highlight the importance of providing multiple opportunities for self-reflection using guided video reflection and checklists for objective self-assessment in the clinical lactation field.

## Background

Midwives are frontline providers of postpartum care, supporting mothers with breastfeeding initiation and maintenance [1, 2]. Nurse-midwifery students report feeling unprepared to support breastfeeding families upon graduation because they receive inadequate training in clinical lactation [3]. Throughout nursing, midwifery, and medical school, there are limited opportunities to actively practice skills because mothers often do not want trainees crowding their rooms and touching their newborns [4]. Healthcare professional students leave their maternal-child rotations with experience as an observer, not an active healthcare provider [4].

High-fidelity simulation is the ideal learning modality for technical and non-technical skills acquisition and transfer to patient care [5, 6]. The objective structured clinical exam (OSCE) is an approach where a standardized patient actor interacts with a health professional student in a mock clinical scenario. The clinical case scenario (conceptual fidelity) combined with the realistic nature of the clinic room and mock patient (psychological fidelity) enables the student to suspend disbelief and practice clinical skills in a way that leads to improved learning outcomes [7, 8]. A breastfeeding-related OSCE has been described in midwifery education, but only with low-fidelity breast models (physical fidelity) [9–11]. Best practices in lactation simulation have not been defined.

An OSCE in clinical lactation (LactSim OSCE) with all three elements of fidelity including conceptual, psychological, and physical is now possible due to the development of a high-fidelity Lactation Simulation Model (LSM) [8, 12]. We developed a hybrid, high-fidelity LactSim OSCE where nurse-midwifery students took turns role-playing as a breastfeeding patient by wearing the LSM. The primary objective of this pilot study was to provide participants multiple opportunities for reflecting in and on practice to determine and compare participants’ perceived self-confidence in clinical lactation skills to their clinical performance in the LactSim OSCE. The secondary objective was to describe the feasibility of implementing the LactSim OSCE within a midwifery curriculum by describing preparatory materials, logistics, and time spent on tasks during the OSCE.

## Methods

### Overview of study and study participants

Nurse-midwifery graduate students (*N* = 15) were enrolled in regularly scheduled coursework. In September of their final year, study participants were exposed to basic and advanced breastfeeding management topics through two simulation-based workshops consisting of lectures with integrated hands-on activities with high-fidelity LSMs (Grabowski A, Anderson OS, Chuisano SA, Zielinski RE, Hammer L, Sadovnikova A: Integrating high-fidelity lactation simulation into the nurse-midwifery classroom, in preparation). Study participants completed the LactSim OSCE in January prior to starting their final clinical rotations. Investigators held a focus group in April to obtain participant’s feedback on the September workshops and LactSim OSCE. Consent was obtained from 12 of the 15 midwifery students for retrospective analysis of collected data. Participants were not required to complete all study activities and surveys. The University Institutional Review Board approved the secondary analysis of existing data (HUM00148905).

### LactSim OSCE framework

Three LactSim OSCEs were developed to represent common breastfeeding challenges that could occur within the first 6 weeks postpartum and fall under the domain of midwifery care and align with the USBC and IBLCE core competencies for health professionals (Table 1, Case 3 in Supplement 1) [13, 14]. The LactSim OSCE was designed for either a trained actor (standardized patient) or a student learner to play the role of the breastfeeding mother.

**Table 1:** LactSim OSCE cases used in pilot study

Case 1	Case 2
**Chief complaint:** Breast heaviness and not enough milk	**Chief complaint:** Not enough milk
**Materials:** LSM, newborn baby doll, spoon for colostrum collection	**Materials:** LSM, breast pump
**Clinician’s learning objectives** 1. Perform a breast exam to identify engorgement, plugged ducts, and nipple anatomical variations that explain the patient’s presentation and chief complaint 2. Teach at least 2 massage techniques for engorgement 3. Describe in layman’s terms the physiology of engorgement 4. Discuss strategies to manage engorgement 5. Hand express ½ teaspoon of colostrum into spoon 6. Observe the patient hand express ½ teaspoon of colostrum 7. Teach “cross-cradle” asymmetric latch position on the right 8. Discuss infant weight loss significance and feeding options (supplementation)	**Clinician’s learning objectives** 1. Assess patient’s goals for breast milk production and infant feeding 2. Provide strategies for increasing milk production 3. Perform breast exam 4. Assemble a breast pump 5. Identify correct flange size for both breasts 6. Demonstrate hands-on-pumping massage techniques 7. Observe the mother assembling the pump and performing hands-on-pumping 8. Describe 2 ways to promote efficient pumping 9. Describe milk storage conditions

### Preparation for the LactSim OSCE

Most participants engaged in a voluntary practice session co-facilitated by two International Board-Certified Lactation Consultants (IBCLC) a week prior to the LactSim OSCE. The investigators developed a packet of preparatory materials (Supplement 2) with suggested readings, learning objectives, patient and clinician scripts and cue cards, and objectives and tasks for the learner in the clinician’s role. Two of the study investigators, one acting as a patient and one as a clinician, filmed a series of instructional videos for each case [15]. All preparatory materials were intended to be made available to the participants a week before the LactSim OSCE. The participants were told that the LactSim OSCE would not be graded and that they should prepare for all three cases and that they would be randomly assigned to play the role of clinician or patient for only two of the three cases. Participants were aware that in-room facilitators would observe the encounter and provide individualized feedback during the debrief.

### LactSim OSCE design

Upon arrival at the simulation facility, participants completed a self-efficacy questionnaire (Table 2) to report their confidence in clinical lactation skills (Pre-test). Participants were randomly divided into pairs and then assigned to begin in either room A (case 2) or B (case 1). Participants had 10 min to complete each case followed by 5 min of feedback from the in-room facilitator. Each pair then switched to the opposite room and switched roles. All encounters were audio-video recorded using built-in technology in the simulation facility. Immediately after both cases, participants completed the self-efficacy questionnaire (Post-test) and an encounter evaluation questionnaire (Table 2). Within a month of the OSCE, participants watched the case in which they role-played as a clinician and completed the “Reflection on Practice” questionnaire (Table 2). Video is increasingly being incorporated into high-fidelity simulation scenarios as a method for students to reflect on their performance and further develop clinical judgement [16].

**Table 2:** Summary of outcome measures and evaluation tools

Outcome	Evaluation tool	Timepoint	Item, administration, scoring
Self-reported confidence in clinical skills relevant to lactation support	Self-efficacy questionnaire	Immediately prior to and after LactSim OSCE	● Perceived confidence in clinical lactation skills ● 28 items [close-ended] ● Defined, 7-point Likert scale
Participants’ preparation for and satisfaction with the LactSim OSCE experience	Encounter evaluation	Immediately after LactSim OSCE	How did you prepare for the LactSim OSCE? [multiple choice, close-ended] Agreement with statements on appropriateness of case scenario content, expectations, time, in-room facilitator feedback, and equipment ● Nine questions [close-ended] o Defined, 7-point Likert scale Four questions [open-ended]
Self-assessment of clinical performance	“Reflection on Practice” questionnaire–technical skills checklist	Within a month of the LactSim OSCE	Clinical skills a clinician should perform during the LactSim OSCE ● 21 questions [close-ended] ● Yes, no, cannot see, cannot hear, not applicable to my case, unsure
Self-reflection on successes and areas of improvement related to clinical performance	“Reflection on Practice”—written reflection	Within a month of the LactSim OSCE	Two questions [open-ended] ● Description of a moment of success in clinical performance (with time stamp) ● Description of a moment where improvement in clinical performance is warranted (with time stamp)
Perceived overall ability to perform clinical skills relevant to lactation support	“Reflection on Practice”—perceived overall competency	Within a month of the LactSim OSCE	● Agreement with statement on overall ability to perform breastfeeding skills ● Defined, 7-point Likert scale

### Relationship between participants’ self-efficacy and clinical performance

The self-efficacy questionnaire was modeled after similar questionnaires used in breastfeeding education [17, 18]. Four investigators with clinical lactation and maternal care experience developed and reviewed the technical skills checklist. Both the self-efficacy questionnaire and the technical skills checklist were assessed by four investigators for face and content validity [19]. Within a month of the LactSim OSCE, participants completed the “Reflection-On-Practice” questionnaire which consisted of a technical skills checklist (Table 3) with skills a clinician should perform during the LactSim OSCE (68 items, case 1; 42 items, case 2), a pre-post rating of perceived competence in clinical skills, and a written self-reflection. Participants used the checklist to follow along as they watched the audio-video recording of their performance as the clinician and document when they saw or heard themselves complete a predefined clinical skill. Before and after watching their video, participants were asked to rate their perceived competence in performing breastfeeding skills. To reflect on practice, participants answered two open-ended questions where they had to select with a timestamp and then elaborate upon a specific moment during which they performed breastfeeding skills well and another moment where they recognized a need for improvement. Participants were able to provide feedback about any topic at the end of the questionnaire via an open-ended question: “Anything else you’d like to add?” Answers to the open-ended questions were summarized via thematic analysis.

**Table 3:** Technical skills checklist used by the participants as part of the “Reflection on Practice” questionnaire

Questions	Description	Points possible	Case
General	Handwashing, permission, draping, no jargon	4	Both
Breast assessment	Inspection and palpation	5	Both
Breast massage	Reverse pressure softening, lymphatic drainage, other massage technique not listed*	3	Both
Hand expression	Demonstration and watched patient	2	Case 1
Infant positioning and attachment at the breast	Skin-to-skin, tummy-to-tummy, stimulate baby’s mouth	3	Case 1
Breast pump setup and use	Assembly, flange size, centering nipple, removal	4	Case 2

*Technique utilized only in case 2

An investigator acting as an independent rater watched the recordings and used the technical skills checklist to rate participants’ performance. The rater’s responses were then compared directly to each student’s response in the “Reflection on Practice” questionnaire, and the results were summarized as a percent agreement between the rater and study participant. When participants answered that they could hear themselves performing the skill, but the performance of the skill could not be visualized due to technical limitations, the rater counted this as “yes.” If the participant responded that the skill was not applicable to the case or they were “unsure” if the skill were applicable, the rater categorized these responses as “no.”

### Evaluation of the feasibility of the LactSim OSCE

In the encounter evaluation questionnaire, participants answered questions about how they prepared for the LactSim OSCE, how to improve the role-playing experience, and whether case scenarios, expectations, allotted time, in-room facilitator feedback, and equipment were adequate. A codebook (Supplement 3) was developed based on the technical skills checklist by three investigators to quantify key themes found in the open-ended questions. Two investigators independently coded the responses. Disagreements were discussed and rectified.

Time on task can be used to assess feasibility of completion of the simulation in the time provided as well as thoroughness of task completion [20]. By determining the amount of time (measured in seconds) students spend on each task within the simulation, investigators can ensure sufficient time is provided and develop a framework for the number of simulation hours required in clinical lactation to transfer skills to patient care. Time spent per task was assessed by two investigators by viewing the audio-video recordings and using a codebook (Supplement 5) to document the time it took for case set-up, interaction between participants during the case (encounter), including engagement with the LSM, interaction between participants and facilitator, and 5-minute feedback session (debrief). Engagement with the LSM was adapted from the “Technical Skills Checklist” and further defined as any interaction between participants or facilitator and the LSM, including utilizing one’s hands directly on the LSM for skills like hand expression, attaching a tool like a breast pump or newborn doll to the LSM, or pointing to the LSM as one would occur during the visual inspection portion of a breast examination. An investigator served as the patient-actor for one of the participants due to the odd number of students; this participant’s video was excluded from analysis and instead used to make iterative improvements to the codebook. After the video codebook was developed, two investigators watched the cases to code each action and interaction, making note of the time (minutes and seconds) per action and interaction. Agreement was analyzed for each case overall, by dyad and by theme. Percent agreement above 90% was deemed acceptable [21]. Facilitator’s interjections and questions posted by participants outside of their role as a clinician or patient were transcribed and analyzed thematically for trends.

A focus group was included as part of a sequential explanatory mixed-methods approach in which the qualitative data from the discussion would help to supplement and interpret survey responses [22]. The focus group, conducted in a classroom setting, provided a neutral space for the participants to articulate what worked well, what could be improved, and expand upon their experience with the LactSim OSCE with an impartial facilitator. An investigator served as the notetaker and did not interact with the participants during the session. The session was not audio-video recorded due to lack of an available recording device to suit the focus group room. Three investigators from the study team reviewed data from the LactSim OSCE to develop discussion questions for the focus group (Supplement 3).

### Statistical analysis

Study participants were not required to complete study documents or participate in study activities. Data were analyzed in aggregate, when appropriate. Statistical analyses were performed in GraphPad Prism (V8.3) and Excel with the XLSTAT package. Descriptive statistics were used to determine the mean and standard deviation and to summarize findings from the qualitative data codebooks. To determine the reliability and consistency of instruments, Cronbach’s α and percent agreement were calculated. Factor analysis could not be performed due to sampling inadequacy. Only items 1–8, 10, 11, 13–15, 20, 21, 27, and 28 from the self-efficacy questionnaire were included in data analysis as they aligned directly with the learning objectives for cases 1 and 2 (Supplement 6). A Wilcoxon matched-pairs signed rank test was used to measure the change in self-efficacy.

## Results

### Study participants

Data from nine female participants, all in their second year of the nurse-midwifery program, were included in the final analyses. Five participants had at least some prior clinical or personal breastfeeding experience. All participants had performed at least one breast examination, provided breastfeeding education to patients, and utilized the LSM during the September simulation-based workshops in a classroom setting (Grabowski A, Anderson OS, Chuisano SA, Zielinski RE, Hammer L, Sadovnikova A: Integrating high-fidelity lactation simulation into the nurse-midwifery classroom, in preparation). One student was removed from all analyses, as she did not have a partner for the OSCE and talked through the case instead of performing the skills. Additional demographic information has been described elsewhere [12].

### Participants’ overall feedback on the LactSim OSCE experience

Nine participants (*N* = 9/9) reported that the case scenarios were “just right” based on their current breastfeeding skill and knowledge level. One participant wrote that the LactSim OSCE was a “great experience overall” and another participant wrote, “Thank you for allowing me to take part in this wonderful experience.” One student indicated that she would benefit from watching the preparatory videos again. The participants agreed (5.9/7) the expert in-room facilitator provided tangible and realistic suggestions for improvement. The participants (*n* = 7) indicated that the LSMs are made very well, improved the realism of the encounter (5.7/7), and they were key for supporting hands-on practice in clinical lactation [12]. During the focus group, the participants indicated that role-playing is a great modality for learning because of the ability to practice being face-to-face with a mock breastfeeding patient.

### Participants’ immediate feedback on the LactSim OSCE experience

Three main themes emerged from the mixed data within the evaluation questionnaire: more preparation, more time for the case, and clearer expectations. Participants (*n* = 7/9) reported that they prepared for the OSCE by reading through the cases and guidelines. Many of the participants attended the voluntary practice session (*n* = 6/9) the week prior, and one participant practiced the scenarios with a classmate ahead of time. The majority of participants (*n* = 5/9) wanted “better background on how to present as the patient” and “better acting skills,” highlighting the need to better prepare participants to serve as “mock patients” in simulated scenarios. Most participants (*n* = 6/9) wrote that “it is important to allow for more time [because it] was difficult to accurately discuss and educate a patient on so much in that time frame.” Some participants (*n* = 3/9) were “unclear about the objectives” of the LactSim OSCE.

### Participants’ reflection-on-practice

Four participants from case 1 and three from case 2 completed the technical skills checklist within the “Reflection on Practice” questionnaire. Participants reported low confidence (4.4/7) in their breastfeeding skills ability upon beginning the questionnaire, with no change (4.4/7, *P* > .9) following the reflection-on-practice exercise. In case 1, each participant (*n* = 4/4) noted something different when asked to select a moment when they performed a clinical skill well: (1) “summarized lymphatic massage, spoon feeding, positions of baby,” (2) “described hand expression well, did well with correcting mother’s hand expression technique,” (3) “Assessing the patient’s complaint difficulty latching/flat nipple,” and (4) “reverse pressure softening.” When asked to select an area for growth, most participants (*n* = 3/4) highlighted that they did not perform a complete breast examination.

When reviewing case 2, two participants wrote that they demonstrated pumping and flange sizing well, and one participant reported that she did a good job gathering the patient’s history and discussing the patient’s milk supply. When asked to reflect on what could have gone better, all participants provided different responses: (1) “I didn’t really notice the shape of the nipple and how that may be related to how deep or shallow the latch of the baby may be. [Facilitator] pointed this out to me. I did a breast assessment at around 9:11, but I realize it was not as thorough as it should have been. I did not palpate to the axilla, or the shape of the nipples,” (2) “While performing the breast exam I forgot to do a full exam of the nipples. I also felt that I ran out of time and did not have time to show the patient how to fully put together the breast pump and use it hands on.” (3) “In retrospect, I could have gone through a couple different feeding positions with mom to ensure proper hold and latch.”

Overall, there was adequate agreement between the independent rater and the student for case 1 (49/68, 72.06%) and for case 2 (29/42, 69.05%) as to which clinical lactation skills were performed during the LactSim OSCE. The majority of the disagreement (68.75%, 22 of the 32 total instances of disagreements) occurred when the student indicated they had completed the skill, while the rater disagreed. The skills with the most disagreement in both cases were related to breast assessment (*n* = 15/32) (Supplement 7).

### Relationship between perceived self-efficacy and clinical performance

There was a discrepancy between perceived self-efficacy immediately before and after the LactSim OSCE and the participants’ self-assessment of their performance during the guided video reflection exercise. Participants’ (*N* = 6) confidence in clinical lactation was high before (5.4/7) and after (5.6/7) completing the LactSim OSCE. Participants (*N* = 7) reported lower confidence (4.4/7) in their overall breastfeeding skills immediately before and after completing the “Reflection on Practice” exercise which better approximated their self-assessment of their performance.

For example, the average score for self-efficacy in breast assessment was 6.25/7 (range 5–7 for *n* = 4 participants), suggesting a high degree of confidence in that skill. Three of those four participants wrote that they needed additional practice with breast assessment in their “Reflection on Practice” questionnaire, with comments such as: (1) “could have done a better job doing a more thorough breast examination at the beginning and examining both nipples better,” (2) “really the whole video but especially here I didn’t address the flat left nipple,” (3) “Should have completed a thorough breast [examination] with inspection and palpation. Did not palpate the patient’s breast.”

### Evaluation of the LactSim OSCE feasibility and instrument validity

**Fig. 1:**
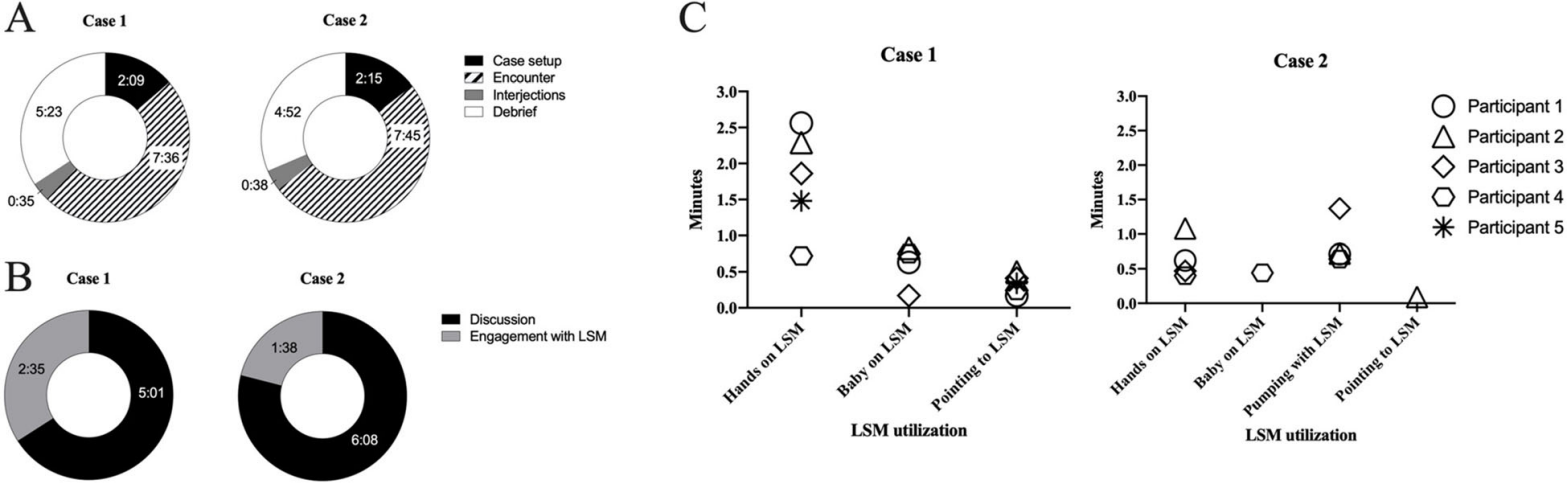
Utilization of time during the LactSim OSCE. a Time spent on case set up, interruptions, encounter, and debrief. b Time spent utilizing the LSM during the encounter. c LSM utilization by subcategory

Four co-authors agreed that 10 min would be sufficient for the practice of the indicated tasks in each case (Table 1) as the participants had significant prior exposure to breastfeeding management and simulation-based practice (Grabowski A, Anderson OS, Chuisano SA, Zielinski RE, Hammer L, Sadovnikova A: Integrating high-fidelity lactation simulation into the nurse-midwifery classroom, in preparation). Participants were not able to utilize the full 10 min for each LactSim OSCE case because they spent the first 2:09 min and 2:15 min of cases 1 and 2, respectively, discussing the set-up and logistics (Fig. 1a). While facilitators were only meant to observe, the cases were interrupted several times either by the in-room facilitator or the participants, with interruptions totaling 35 and 38 s for case 1 and case 2, respectively. After accounting for the setup and interruptions, the participants had an average of 7:36 and 7:45 min to complete the encounter in case 1 and case 2, respectively. Participants in case 1 spent 34% (2:35 min) of the encounter engaging with the LSM while participants in case 2 touched, pointed at, or used the LSM for 21% (1:38 min) of the total case time (Fig. 1b**)**. There was significant variation in the amount of time spent on each breastfeeding skill between participants (Fig. 1c).

The conversations between facilitators and participants during case interruptions further emphasized the importance of adequate student preparation for the role of a mock patient. The facilitator in case 1 assumed the role of a “support person” on six occasions, with comments like: “I’m just concerned about how she is going to manage all of this, all this hard breast situation, what is she going to do about that?” and “What is she going to do with that flat nipple on that other side?” The majority of the facilitator’s interruptions in case 2 were to help (*n* = 4/9) with logistics, such as setting up the breast pump and providing clarification (*n* = 4/9) of the case goals when participants appeared to be unsure. The most common (*n* = 7/13) reason for interruptions initiated by participants in both cases was to ask the facilitator a clarifying question about the LSM’s feature, such as, “Is she supposed to have plugged ducts because I feel some in here?”

Four investigators agreed that the self-efficacy questionnaire and the technical skills checklist had correct content and were appropriate for the level of the learner. The self-efficacy questionnaire had a high degree of internal consistency as the Cronbach’s α was > 0.87 even after a question was removed at random. The qualitative data codebooks for both the encounter evaluation questionnaire and “Reflection on Practice” questionnaire were reliable as the inter-rater agreement between the two coders was respectively 90.4% and 94.4%. The codebook developed for audio-video recordings was used with a high degree of agreement between raters (case 1 91.1%, case 2 94.4%).

## Discussion

This pilot study is the first description of a high-fidelity OSCE in clinical lactation with all three components of fidelity in which health professional students role-play as either the clinician or the breastfeeding patient. A strength of this study is that nurse-midwifery students had multiple opportunities for self-reflection in and on their LactSim OSCE experience, supporting higher engagement and learning [23–25]. We are the first to describe how long a learner spends on various tasks relevant to an encounter with a breastfeeding patient in a simulation-based training exercise. Time spent on task has been reported for breast assessment simulations and is important to consider when designing effective training exercises [26, 27]. The ultimate goal would be to establish how much time to dedicate to the practice of each clinical lactation skill in a simulated encounter for learners to then translate that skill to the care of breastfeeding patients to improve health outcomes.

The LactSim OSCE approach was similar to the lactation and infant feeding OSCE described by Muldoon et al., where nurse-midwifery students had a voluntary preparatory session prior to the OSCE and during the OSCE students sequentially completed two scenarios each of 10-min duration while acting as either the clinician or the breastfeeding patient [9]. In contrast with our work, a breast model was not used in the OSCE described in Muldoon et al., and students disagreed that the lactation and infant feeding OSCE reflected a real-life clinical scenario [9]. Moreover, learners in the Muldoon et al. work were not provided with opportunities to reflect upon their clinical performance [9].

Previous work suggests that a high self-efficacy in clinical lactation skills is common among nurse-midwifery students in the final year of their training program [28]. While self-efficacy in clinical skills is a standard metric in high-fidelity simulations, our findings indicate that perceived self-efficacy was not a reliable outcome metric of immediate learning gains from the LactSim OSCE [29]. The participants’ high perceived self-confidence in breast assessment, hand expression, and newborn positioning and attachment in the immediate period following the LactSim OSCE did not align with the self-assessment of their clinical performance in the “Reflection-on-Practice” exercise. The Dunning-Kruger effect is evident when healthcare professionals and trainees overestimate their abilities, highlighting the importance of including outcome measures other than self-efficacy when evaluating an educational intervention [30, 31].

When a trainee self-assesses his or her level of competency in simulation-based training experiences, the result is a highly reliable and valid educational outcome [23]. Our findings underscore the importance of providing multiple opportunities for self-reflection using guided video reflection and checklists for objective self-assessment in the clinical lactation field [23, 32, 33].

### Limitations

A limitation of our study was the small sample size, lack of completion of study materials by participants, and significant time limitation during the OSCE. Due to technical difficulties, the study participants did not receive the preparatory materials until the night before the LactSim OSCE which limited the amount of time they had to prepare. The nurse-midwifery students in our study may have felt more prepared to serve as the clinician and breastfeeding patient if they had access to the preparatory videos and materials earlier, but in previous work, early access to preparatory materials did not align with perceived feelings of preparedness [9]. Interruptions by in-room facilitators to help move the scenario along are not uncommon in the simulation literature, further underscoring the need for adequate preparation by each student, instructors, and facilitators [20]. The audio-video recording capability within the simulation facility did not allow for a full view of the clinic room. In future studies, two camera angles could be used: one facing the patient and one from behind and above the patient’s head so that the skills performed on the high-fidelity breast model can be visualized clearly without the clinician’s body blocking the camera.

## Conclusion

In this sequential, explanatory, mixed-methods study we describe the first example of an OSCE in clinical lactation (LactSim OSCE) with all three components of fidelity where nurse-midwifery students role-played as either the clinician or the breastfeeding patient by wearing a high-fidelity breast model. An important contribution of this work to the field of breastfeeding education is the development of a framework for how to evaluate clinical lactation skills in a simulation exercise and the documentation on time spent per task by the students. The documentation of time spent per task serves as preliminary guidance for clinical faculty and simulation staff on how to develop and execute a lactation simulation scenario [20]. The Reflection-on-Practice questionnaire provided a safe space for each participant to view and evaluate their clinical performance in the LactSim OSCE and identify targeted areas for improvement. Study participants experienced significant gains in clinical knowledge relevant to lactation support due to multiple opportunities provided by the investigator team for written and oral reflection in and on practice.

## Supplementary information

**Additional file 1: Supplement 1.** Case 3 Learning Objectives.

**Additional file 2: Supplement 2.** Prep Materials.

**Additional file 3: Supplement 3.** Open Ended Questions Codebook.

**Additional file 4: Supplement 4.** Focus Group Discussion Questions.

**Additional file 5: Supplement 5.** Video Codebook

**Additional file 6: Supplement 6.** Self-Efficacy Questions by Case.

**Additional file 7: Supplement 7.** Disagreements between participants and independent rater in Technical Skills Checklist.

## Data Availability

The datasets used and/or analyzed during the current study are available from the corresponding author on reasonable request.
