# Supplementary material for: Midwifery students better approximate their self-efficacy in clinical lactation after reflecting in and on their performance in the LactSim OSCE
Source: Adv Simul (Lond). 2020 Oct 23;5:28. doi: 10.1186/s41077-020-00143-z (PMC7583289; doi:10.1186/s41077-020-00143-z)
Supplement: Supplementary file 1 — Additional file 1: Supplement 1. Case 3 Learning Objectives. [file 41077_2020_143_MOESM1_ESM.docx]

| **Chief Complaint:** *Breast Pain* |
| --- |
| **Materials:** Essential LSM |
| **Clinician’s learning objectives**   1. Obtain focused history of the breast pain 2. Perform breast exam 3. Talk through your differential diagnosis for breast pain with the patient and explain your rationale for your top diagnosis. 4. Describe next steps in management 5. Obtain milk for culture via hand expression 6. Ensure that patient understands management plan |
