# Supplementary material for: Midwifery students better approximate their self-efficacy in clinical lactation after reflecting in and on their performance in the LactSim OSCE
Source: Adv Simul (Lond). 2020 Oct 23;5:28. doi: 10.1186/s41077-020-00143-z (PMC7583289; doi:10.1186/s41077-020-00143-z)
Supplement: Supplementary file 2 — Additional file 2: Supplement 2. Prep Materials. [file 41077_2020_143_MOESM2_ESM.docx]

**UM Nursing Midwifery - LiquidGoldConcept LSM Trial**

**January 11, 2018 Skills Assessment**

**Instructions:**

*When:* January 11, 2018 between 1 and 4 PM

*Where:* UM Nursing Sim Center

*What:*

- This experience is not graded. The Clinician-Patient Dyad Simulated Encounter is meant to prepare you for your clinical rotations.
- You must prepare all three cases.
- On the day of the skills assessment, you will be randomly assigned to complete two cases, one as the clinician and another as the patient.
- Dr. Lisa Hammer and Melisa Scott will serve as in-room evaluators.
- You have 10 minutes to complete the entire encounter.
- You will receive 2-3 minute of feedback from the in-room evaluator.

**Suggested method of preparation**

1. Read the cases and guidelines (below)
2. Watch the example videos for each case
   1. Notes:
      1. Case 1 video has the most comprehensive breast exam--follow this structure for all the cases
      2. Case 2 video has the most comprehensive pumping instructions--disregard the fact that, in the video, the patient goes from wearing an essential to wearing an advanced LSM.
      3. The videos are longer than 10 minutes, that’s because we tried to show the techniques from far away and close up. Your goal is to run through each case in 10 minutes.
3. Read the articles recommended for each case.
4. Practice being the clinician and the patient with a friend!

**Recommended Readings:**

We recommend that you use these resources to prepare for each case.

**Case 1**

- Engorgement Protocol #20 <http://www.bfmed.org/protocols>
- Physiology of Lactogenesis 2: <http://jn.nutrition.org/content/131/11/3005S.long>

**Case 2**

- Human Milk Storage Protocol #8 <http://www.bfmed.org/protocols>
- Principles for maintaining or increasing breast milk production: <https://www.ncbi.nlm.nih.gov/pubmed/22150998>

**Case 3**

- Breast pain and mastitis protocols #4 and $26 <http://www.bfmed.org/protocols>

**Case 1 Chief complaint: Breast heaviness and “not enough milk”**

The patient will be wearing the Advanced LSM. Please, engorge both breasts before beginning the case. Focus on nipple size/shape, plugged ducts, and engorgement. While the Advanced LSM has a lot of other features, for the purposes of this case, disregard the surgical scars, ectopic tissue, and abscess.

**Patient’s Cue Card (Jane Smith):** You are a first time mother, 25-years-old, on day three postpartum and are ready to be discharged from the hospital. You vaginally delivered baby Sam (2750 g) at 36 2/7 weeks gestation. You read about the benefits of breastfeeding and want to exclusively breastfeed for 6 months. The baby last ate 6 hours ago. You are happy that your baby is sleeping through the night. This morning, baby is 72 hours old and weighs 2450g (weight loss of 10%). You woke up with hard and swollen breasts with small lumps in them. So far, you’ve only latched the baby cross-cradle. You are worried that you are not producing enough milk because the baby doesn’t seem to be satisfied at the breast. You think Sam latches well on the right--you’ve seen the baby’s whole mouth over the nipple. On the left breast, Sam tends to slip off. One of the doctors told you this morning that you will need to start supplementing with formula because Sam’s weight loss is significant.

**Clinician’s Cue Card:** Obviously, you have read the patient’s cue card, so you don’t need to gather all of that history again. This exercise is meant to simulate a real patient encounter with the goal of helping you assess your comfort with brief history taking, breast exam, breastfeeding techniques, and counseling. Please, introduce yourself, wash hands, and, if necessary, obtain additional information about obstetric, labor, and postpartum history as it may relate to the breastfeeding experience. Ask permission prior to doing a procedure. As you are performing the breast exam, explain your findings to the patient. Don’t use jargon when explaining physiology, risk factors, and your management plan. Make sure that the patient can replicate the skills by observing the patient perform massage and hand expression.

**Pertinent positives and negatives on physical exam:** Temperature, heart rate, respiratory rate, and blood pressure for mother and child are within normal range. The mother has a large, bulbous nipple on the right and a flat nipple on the left. Infant has a small mouth, but does not have ankyloglossia or lip-tie. Infant had one bowel movement on day one of life, two bowel movements on day two of life, and one bowel movement on day three of life.

**Your goals as the clinician are the following:**

1. Perform a breast exam to identify engorgement, plugged ducts, and nipple anatomical variations that explain the patient’s presentation and chief complaint
2. Teach at least 2 massage techniques for engorgement
3. Describe in layman’s terms the physiology of engorgement
4. Discuss strategies to manage engorgement
5. Hand express ½ teaspoon of colostrum into spoon
6. Observe the patient hand expression ½ teaspoon of colostrum
7. Teach “cross-cradle” asymmetric latch position on the right.
8. Discuss infant weight loss significance and feeding options (supplementation)

**Detailed Guidelines for Clinician**

1. Perform breast exam to identify engorgement, plugged ducts, and nipple anatomical variations
   1. Disregard abscess, ectopic tissue, milk bleb, and surgical scars. These features are not necessary for this case.
   2. A breast exam in a lactating patient is slightly different than in a non-lactating patient (as in the case of breast cancer screening). You do not need to lie your patient down all the way. You must visually inspect both breasts *simultaneously*. You may drape the patient appropriately when you move on to palpation. You must inspect and palpate axillae and breast tissue (including underneath the breast) to receive full points.
   3. Explain to the patient how her nipple anatomy may influence her newborn’s latch.
2. Describe in layman’s terms the physiology of lactogenesis 2 and engorgement (i.e. what is causing the breast fullness)
3. Demonstrate breast massage techniques to alleviate engorgement
   1. choose 2 out of 3 listed below
      1. Lymphatic drainage
         1. Option 1. Hold breast with both hands and slide breast tissue over pectoralis major muscle (3x to axillary and 1x to sternal lymph nodes)
         2. Option 2. Lightly sweep hands over breast tissue toward axilla
      2. Reverse pressure softening
         1. Option 3. Use pads of fingers to compress areolar tissue towards chest wall (hold for 30 seconds on a real patient to observe dimpling of edematous skin)
   2. Ensure that your patient can replicate the techniques on herself.
4. Demonstrate hand expression of colostrum
   1. Position index finger and thumb (distal phalanges) opposite each other approximately where a newborn would deeply latch on the breast tissue. *Do not glide or slide* your fingers over the breast tissue as that may bruise or damage skin and is not an effective technique.
   2. Express approximately ½ teaspoon
5. Ensure that your patient can replicate the hand expression technique on herself
6. Discuss various strategies to manage engorgement
   1. Hot vs cold compresses
   2. Frequent feeding
   3. Massage
   4. Pumping
   5. NSAIDs
7. Teach the patient how to do a “cross-cradle” asymmetric latch on the left.
8. Propose a feeding plan
   1. Determine whether supplementation with formula or breast milk is warranted.
      1. If yes, how will you supplement? Syringe feeds, supplemental nursing system, spoon/cup feeding, bottle feeding.
      2. Does her nipple anatomy change your feeding plan? Pumping vs feeding?
   2. Discuss timing for feeds (how often and how long)

**Case 2. Chief Complaint: “Not enough milk”**

The patient will wear the Essential LSM. Disregard the mastitis.

**Patient’s Cue Card (Janet Brown):** You are a 35-year-old working mother of two. You have a three year old and a 5 week old named Alex who was born at term via c-section. You have to go back to work in 3 weeks and you are worried about maintaining milk production. Your partner works full-time and both the toddler and newborn will be at daycare. You’ve had a good breastfeeding experience so far and have been supplementing with formula only at night because your friend told you that would help the baby sleep better. Two days ago, a lactation consultant assessed milk transfer by weighing the newborn before and after a feed on both breasts--1 ounce was transferred.

**Clinician’s Cue Card:** Obviously, you have read the patient’s cue card, so you don’t need to gather all of that history again. This exercise is meant to simulate a real patient encounter with the goal of helping you assess your comfort with brief history taking, breast exam, breastfeeding techniques, and counseling. Please, introduce yourself, wash hands, and, if necessary, obtain additional information about obstetric, labor, and postpartum history as it may relate to the breastfeeding experience. Ask permission prior to doing a procedure. As you are performing the breast exam, explain your findings to the patient. Don’t use jargon when explaining physiology, risk factors, and your management plan. Make sure that the patient can replicate the skills by observing the patient ‘hands-on’ pumping.

**Pertinent positives and negatives on physical exam:** Temperature, heart rate, respiratory rate, and blood pressure for mother and child are within normal range. After observing a feed on the left, you see that the left nipple comes out slanted/smushed. Disregard the mastitis coloration on the left. Infant growth is within normal range: Alex’s birth weight: 3650 g, Alex’s weight today: 4650 g

**Your goals as the clinician are the following:**

1. Assess patient’s goals for breast milk production and infant feeding
2. Provide strategies for increasing milk production
3. Perform breast exam
4. Assemble a breast pump and identify correct flange size for both breasts
5. Demonstrate hands-on-pumping massage techniques
6. Observe the mother assembling the pump and performing hands-on-pumping
7. Describe 2 ways to promote efficient pumping
8. Describe milk storage conditions

**Detailed Guidelines for Clinician (Essential w/ normal nipples):**

1. Assess patient’s goals for breast milk production and infant feeding
   1. How much milk does the patient wish to produce and for how long?
   2. Discuss the importance of regular pumping/stimulation to maintain milk production
   3. Provide education on infant sleep patterns and pros/cons of formula feeding at night as it relates to milk production
2. Perform breast exam
   1. Disregard mastitis.
   2. A breast exam in a lactating patient is slightly different than in a non-lactating patient (as in the case of breast cancer screening). You do not need to lie your patient down all the way. You must visually inspect both breasts *simultaneously*. You may drape the patient appropriately when you move on to palpation. You must inspect and palpate axillae and breast tissue (including underneath the breast) to receive full points.
3. Pumping
   1. Assemble a breast pump and identify correct flange size for both breasts
   2. Demonstrate hands-on-pumping massage techniques
   3. Observe the mother assembling the pump and performing hands-on-pumping
4. Propose a pumping/feeding plan for the next few weeks to maintain and/or increase milk production
5. Describe 2 ways to promote efficient pumping
   1. Looking at baby pictures/videos
   2. Being ‘hands on’
   3. Double-pumping
   4. Massage
   5. Timing
   6. Pumping bras
6. Describe milk storage conditions

**Case 3 Chief Complaint: Breast pain**

The patient will wear the Essential LSM.

**Patient’s Cue Card (Martha Adams):** You are a two-week postpartum first time mother. Your pregnancy was complicated by obesity and gestational diabetes. You delivered baby Taylor via c-section due to fetal macrosomia. Breastfeeding was not initiated within the first 24 hours and lactogenesis 2 was delayed. You really want to breastfeed for at least six months. For the first week of life, Taylor had a shallow latch and your nipple on the left would bleed every time Taylor latched. Taylor was diagnosed with anterior tongue-tie and had it clipped four days ago. Breastfeeding feels better now and the left nipple is healing. Two days ago, however, you started to experience breast pain on the left. You don’t feel like you have a fever, but you have felt really tired and “achy.”

**Clinician’s Cue Card:** This exercise is meant to simulate a real patient encounter. Please, introduce yourself, wash hands, and obtain a history focused on: obstetric, labor, and postpartum as it may relate to the breastfeeding experience. Ask permission prior to doing a procedure. As you are performing the breast exam, explain your findings to the patient. Don’t use jargon when explaining physiology, risk factors, and your management plan.

**Pertinent positive and negatives on physical exam:** Temperature, heart rate, respiratory rate, and blood pressure for child are within normal range. Mother’s heart rate, respiratory rate and blood pressure are within normal range, but her temperature is 100.2 degrees Fahrenheit. Mother’s prepregnancy BMI was 34. There is noticeable red discoloration of the left breast tissue in the upper outer quadrant. The left nipple has a healing fissure but no visible scab, bleeding, or pink discoloration. Patient denies burning sensation on nipple. Breast pain is dull, not sharp or shooting. The newborn’s mouth has no sign oral thrush.

**Your goals as the clinician are the following:**

1. Obtain focused history of the breast pain
2. Perform breast exam
3. Talk through your differential diagnosis for breast pain with the patient and explain your rationale for your top diagnosis.
4. Describe next steps in management
5. Obtain milk for culture via hand expression
6. Ensure that patient understands management plan

**Detailed guidelines for clinician**

1. Obtain history of breast pain
   1. Think about the differential diagnosis for breast pain. Make sure to ask questions to help rule in/out whether pain is musculoskeletal, related to an infection (yeast vs bacterial), or due to inflammation from oversupply or plugged ducts.
2. Perform breast exam
   1. Observe the discoloration on left breast.
   2. A breast exam in a lactating patient is slightly different than in a non-lactating patient (as in the case of breast cancer screening). You do not need to lie your patient down all the way. You must visually inspect both breasts *simultaneously*. You may drape the patient appropriately when you move on to palpation. You must inspect and palpate axillae and breast tissue (including underneath the breast) to receive full points.
3. Talk through your differential diagnosis with the patient and explain your rationale for one over the other.
   1. Explain in layman’s terms the risk factors for infectious mastitis
   2. Explain in layman’s terms the signs and symptoms of a yeast infection, explain why what the patient has is likely not a yeast infection.
   3. Explain in layman’s terms the importance of proper positioning of the body to prevent neck and shoulder pain (musculoskeletal causes of breast pain)
   4. Explain in layman’s terms how to differentiation inflammatory (plugged ducts) vs infectious mastitis.
4. Pick **one**, based on your top differential: Describe next steps in management
   1. Mastitis:
      1. If mother presents with fever, red discoloration of tissue, myalgia, and +/- history of nipple trauma, then
         1. Culture milk
         2. Antibiotics (dicloxacillin)
         3. Continue feeding
      2. Hand express breast milk for culture, use sterile technique
         1. Wipe nipple and areola with ethanol
         2. Wear nitrile gloves
         3. Hand express ½ teaspoon, take care to avoid collecting milk that has touched or rolled down the patient’s skin
   2. Yeast infection
      1. If thrush in infant’s mouth and mom has fissuring of skin with pruritic erythematous rash on nipple/areola, then could start with topical nystatin for mom’s nipples and oral nystatin for baby.
      2. Hand express breast milk for culture, use sterile technique
         1. Wipe nipple and areola with ethanol
         2. Wear nitrile gloves
         3. Hand express ½ teaspoon, take care to avoid collecting milk that has touched or rolled down the patient’s skin
   3. Musculoskeletal
      1. If the pain is due to poor body positioning during latch and or pumping, then advise the mother to stretch her shoulders and try “laid-back nursing.”
   4. Non-infectious
      1. If mother does not have fever or erythema of the breast and no nipple trauma then likely it is non-infectious and could be due to oversupply or plugged ducts. Advise massage techniques and gradual decrease in additional pumping and/or nipple stimulation.
5. Discuss options to relieve or prevent sore nipples
   1. All purpose nipple ointment, breast milk on nipple, improve latch, lanolin
6. Ask the patient to reiterate the management plan
