# Supplementary material for: Midwifery students better approximate their self-efficacy in clinical lactation after reflecting in and on their performance in the LactSim OSCE
Source: Adv Simul (Lond). 2020 Oct 23;5:28. doi: 10.1186/s41077-020-00143-z (PMC7583289; doi:10.1186/s41077-020-00143-z)
Supplement: Supplementary file 3 — Additional file 3: Supplement 3. Open Ended Questions Codebook. [file 41077_2020_143_MOESM3_ESM.docx]

| **Encounter Evaluation Open-Ended Questions** | |
| --- | --- |
| **Question** | **Codes** |
| What could be improved about the structure, logistics, execution of the Clinician-Patient Dyad Simulated encounter? | No suggestions |
|  | An answer that indicates that the student wants more practice ahead of time |
|  | An answer that indicates that the student should have better prepared for the scenario |
|  | An answer that indicates that the student wanted more time for the case and/or feedback |
|  | An answer that indicates that the student thought the objectives were unclear/would have liked clearer expectations to be set |
|  | An answer that indicates that the student discussed logistical details of the case scenario or the realisticness of the scenario |
| What Recommendations do you have for the “patient” to create a more realistic clinical encounter? What would you have done differently? | No suggestions |
|  | An answer that suggests that the student would have liked different patient scenarios - for whatever reason |
|  | An answer that suggests that the student thought/indicated that a standardized patient should be used |
| How could the “clinician” improve his/her counseling, physical exam, and breastfeeding skills? What would you have done differently? | An answer that reflects that the student wishes they had more time prior to the scenario to practice |
|  | An answer that indicates that the student thought the objectives were unclear/would have liked clearer expectations to be set |
|  | An answer in which the student discussed fine tuning details of the case scenario (e.g. time, relevance, diversity) |
| Anything else you’d like to tell us? Your feedback will help us make improvements to next year’s curriculum. | An answer that has already been described in another question - clarification, practice, preparation, use of standardized patient, more diversity, etc... |
|  | An answer that shows that the student would have liked a full-day of OSCE style practice instead of few hours |
|  | An answer the highlights that the student would have liked less assessment and more teaching during the workshop |
| **Self-Assessment Final Open-Ended Question** | |
| Anything else you’d like to add? | An answer that has already been described in another question - clarification, practice, preparation, use of standardized patients, more diversity, etc.. |
|  | An answer shows that student didn’t have their video to assess |
|  | An answer that suggests that student enjoyed their experience with the workshop |
|  | An answer that highlights that the student had good conversations with the evaluator |
