# Supplementary material for: Midwifery students better approximate their self-efficacy in clinical lactation after reflecting in and on their performance in the LactSim OSCE
Source: Adv Simul (Lond). 2020 Oct 23;5:28. doi: 10.1186/s41077-020-00143-z (PMC7583289; doi:10.1186/s41077-020-00143-z)
Supplement: Supplementary file 4 — Additional file 4: Supplement 4. Focus Group Discussion Questions. [file 41077_2020_143_MOESM4_ESM.docx]

| *Discussion questions #1.* a) What is the midwife’s role when supporting a breastfeeding patient? b) What should an encounter between a midwife and a breastfeeding patient look like? c) What would the perfect set of hands-off and hands-on skills be for you as a midwife?  This question set was developed in response to a trend among midwifery students to focus on breastfeeding counseling as opposed to hands-on breastfeeding management. |
| --- |
| *Discussion question #2.* What worked well with the September Workshop experience?  This question was developed strategically to share beneficial experiences with the group, to steer the initiation of the discussion away from a platform to complain. |
| *Discussion questions #3.* During the 3-month clinical rotations, were there opportunities to interact with and practice clinical skills with breastfeeding patients? Which aspects, if any, of the September Workshops helped improve your interactions with breastfeeding patients?  This question set was developed for students to describe first-hand accounts of putting theory and simulated-practice from what they learned from the September Workshops, specifically, into real-patient practice. |
| *Discussion questions #4.* a) What could be improved from the LactSim OSCE? b) Think of an educational experience to help you succeed in obtaining breastfeeding management skills, what would this look like? What would you value?  This question set was developed as the final discussion piece after the students had time to reflect back on the entire experience and on how they were cumulatively assessed. These questions offered an opportunity for the students to provide areas of improvement. |
