# Supplementary material for: Midwifery students better approximate their self-efficacy in clinical lactation after reflecting in and on their performance in the LactSim OSCE
Source: Adv Simul (Lond). 2020 Oct 23;5:28. doi: 10.1186/s41077-020-00143-z (PMC7583289; doi:10.1186/s41077-020-00143-z)
Supplement: Supplementary file 5 — Additional file 5: Supplement 5. Video Codebook [file 41077_2020_143_MOESM5_ESM.docx]

| **Theme** | **Definition, “this theme is to be utilized...”** |
| --- | --- |
| Case set-up | when the patient/clinician is reading the case, putting on the LSM, or setting up the room - prior to the introduction of the midwife. This code may be utilized after “introduction” if the case is paused/interrupted due to confusion. |
| Interaction between students during the case | When one student plays the role of the clinician and one student plays the role of the patient. This includes all history gathering, exams, management discussions, pumping and other breastfeeding skills. |
| Interaction between students and facilitator | anytime the student asks the facilitator a question or the facilitator speaks/appears in the camera to help guide the students. |
| Case Debrief | This code is to be utilized after the 10 minute mark (knock on door) when the facilitator discusses the case and other breastfeeding aspects. If the facilitator utilizes the LSM during feedback, it should be co-coded with “facilitator teaches with LSM”. |
| Interaction between students during the case: subthemes | |
| Hands on LSM | when the students hands are physically touching the LSM. This can include breast exam, massage technique, hand expression, or other aspects. Do not code when the patient is merely sitting in the room talking to the clinician holding her breast. This does not include times in which the patient is passively touching the LSM. |
| Pointing to/Referencing LSM | when the students visually inspect or refer to the LSM. This can be during the breast exam or gesturing to various aspects of the LSM. |
| Pumping with LSM/Flange sizing | when the clinician is providing pumping support to the patient. To use this code, the flange must be on the LSM. This can include fitting the flange size to the breast. |
| Latch work with LSM | when the clinician helps the patient with latch/positioning the baby. |
| Facilitator teaches with LSM | when the facilitator provides support either during the case or during feedback period while directly referring to or touching the LSM. |
