# Supplementary material for: Midwifery students better approximate their self-efficacy in clinical lactation after reflecting in and on their performance in the LactSim OSCE
Source: Adv Simul (Lond). 2020 Oct 23;5:28. doi: 10.1186/s41077-020-00143-z (PMC7583289; doi:10.1186/s41077-020-00143-z)
Supplement: Supplementary file 6 — Additional file 6: Supplement 6. Self-Efficacy Questions by Case. [file 41077_2020_143_MOESM6_ESM.docx]

| Self-Efficacy Skills By Case | Case 1 | Case 2 | Case 3 |
| --- | --- | --- | --- |
| 1. perform a breast exam | ✓ | ✓ | ✓ |
| 1. teach hand expression of colostrum | ✓ |  |  |
| 1. teach hand expression of mature milk |  | ✓ | ✓ |
| 1. teach football latch position | ✓ |  |  |
| 1. teach cross-cradle latch position | ✓ |  |  |
| 1. assemble a breast pump and use with a patient |  | ✓ |  |
| 1. identify correct flange/shield size for breast pump |  | ✓ |  |
| 1. demonstrate massage techniques for engorgement | ✓ |  |  |
| 1. demonstrate massage techniques for plugged ducts |  |  | ✓ |
| 1. describe infant feeding requirements for the first month of life | ✓ | ✓ |  |
| 1. distinguish between a shallow and deep latch | ✓ |  |  |
| 1. describe strategies to manage sore or damaged nipples |  |  | ✓ |
| 1. describe breast milk storage conditions |  | ✓ |  |
| 1. describe physiology of lactation | ✓ |  |  |
| 1. identify maternal medical conditions that may negatively impact breastfeeding success | ✓ | ✓ | ✓ |
| 1. describe lactation initiation strategy for C-section versus vaginally delivered infant |  |  | ✓ |
| 1. describe risks for developing infectious mastitis |  |  | ✓ |
| 1. describe signs and symptoms of infectious mastitis |  |  | ✓ |
| 1. describe management plan for infectious mastitis |  |  | ✓ |
| 1. discuss supplementation strategies for infants with poor weight gain | ✓ |  |  |
| 1. Describe how different nipple shapes may affect breastfeeding success | ✓ |  |  |
| 1. demonstrate how to use a nipple shield |  |  | ✓ |
| 1. differentiate between mastitis and breast abscess |  |  | ✓ |
| 1. provide a differential diagnosis for a breast lump in a lactating woman |  |  | ✓ |
| 1. provide a differential diagnosis for breast pain in a lactating woman |  |  | ✓ |
| 1. provide breastfeeding education to pregnant women |  |  |  |
| 1. teach patients breastfeeding skills | ✓ | ✓ | ✓ |
| 1. provide general breastfeeding education to my patients | ✓ | ✓ | ✓ |
