# Supplementary material for: Midwifery students better approximate their self-efficacy in clinical lactation after reflecting in and on their performance in the LactSim OSCE
Source: Adv Simul (Lond). 2020 Oct 23;5:28. doi: 10.1186/s41077-020-00143-z (PMC7583289; doi:10.1186/s41077-020-00143-z)
Supplement: Supplementary file 7 — Additional file 7: Supplement 7. Disagreements between participants and independent rater in Technical Skills Checklist. [file 41077_2020_143_MOESM7_ESM.docx]

| **Skill** | Case 1  n=19 | Case 2  n=13 |
| --- | --- | --- |
| Washed/cleaned hands before touching patient | 1 | 1 |
| Asked permission to perform exam or touch patient | 1 | 2 |
| Appropriately draped patient throughout visit | 2 | 0 |
| Used non-jargon to explain findings to patient as they came up | 1 | 0 |
| Visually examined BOTH breasts simultaneously | 0 | 1 |
| Palpated both axillae | 2 | 1 |
| Palpated underneath breast tissue | 0 | 1 |
| Palpated both nipples | 2 | 1 |
| Palpated areolae | 4 | 2 |
| Hand expressed a teaspoon of simulated milk | 0 | N/A |
| Watched patient hand express a teaspoon of simulated milk | 0 | N/A |
| Made sure that baby doll was "skin-to-skin" during feeding | 0 | N/A |
| Demonstrated how to stimulate baby's mouth and nose with nipple to encourage wide opening of mouth before latch | 0 | N/A |
| Positioned baby's abdomen against patient's abdomen during feed | 1 | N/A |
| Demonstrated reverse pressure softening technique for engorgement management | 1 | N/A |
| Demonstrated lymphatic drainage technique for management of engorgement | 3 | N/A |
| Demonstrated massage technique, but it isn't listed here | 1 | 1 |
| Demonstrated how to remove pump to prevent loss of milk in the flange | N/A | 0 |
| Assembled breast pump | N/A | 1 |
| Selected appropriate flange size | N/A | 1 |
| Centered the nipple in the flange | N/A | 1 |
